# A Diagnostic of Acquired Hemophilia Following PD1/PDL1 Inhibitors in Advanced Melanoma: The Experience of Two Patients and a Literature Review

**DOI:** 10.3390/diagnostics12102559

**Published:** 2022-10-21

**Authors:** Antonio Gidaro, Giuseppe Palmieri, Mattia Donadoni, Lucia A. Mameli, Leyla La Cava, Giuseppe Sanna, Dante Castro, Alessandro P. Delitala, Roberto Manetti, Roberto Castelli

**Affiliations:** 1 Department of Biomedical and Clinical Sciences Luigi Sacco, Luigi Sacco Hospital, University of Milan, Via G.B. Grassi N° 74, 20157 Milan, Italy; 2 Department of Medicine, Surgery and Pharmacy, University of Sassari, Viale san Pietro N° 8, 07100 Sassari, Italy; 3 Departmental Simple Operative Unit Coagulation, Hemostasis Diseases Hospital S Maria Annunziata, Via Enrico De Nicola N° 14, 07100 Sassari, Italy

**Keywords:** acquired hemophilia A (AHA), immune checkpoint inhibitors (ICIs), nivolumab, pembrolizumab, melanoma, Novoseven^®^, rituximab

## Abstract

Acquired hemophilia A (AHA) is a rare bleeding disorder caused by the development of specific autoantibodies against factor VIII (FVIII). Immunotherapy is a recent therapeutic option that targets the patient’s self-tolerance against tumor cells. Because therapeutic effects of the immune checkpoint inhibitors (ICIs) are mediated by enhancing the immune response to restore antitumor immunity, autoimmune-related adverse effects can be seen in up to 80% of patients during treatment and after treatment. A rare hematologic ICIs-related adverse event is AHA. Hereafter we report two cases of AHA developed during anti-PD-1 immunotherapy for advanced melanoma: one secondary to treatment with nivolumab and one secondary to pembrolizumab. Both patients were treated with activated FVII (Novoseven^®^, Novo Nordisk, Bagsværd, Denmark) as hemostatic treatment combined with the eradication of antibodies anti-FVIII obtained with rituximab. In the last few years these drugs have significantly improved the therapeutic armamentarium for the management of AHA. Indeed, while FVIIa has proven to be an effective and safe tool for the treatment of acute bleeding related to FVIII autoantibodies, rituximab is a promising alternative for the autoantibodies’ elimination and the restoration of normal hemostasis. Our finding supports the use of this combination even in AHA secondary to ICIs treatment.

## 1. Introduction

Acquired hemophilia A (AHA) is a rare bleeding disorder due to neutralizing antibodies against factor VIII. Its incidence is approximately 1.4 per million inhabitants’ year [1]. Although uncommon, these autoantibodies are associated with a high rate of morbidity and mortality as severe bleeds occur in up to 90% of affected patients, and the mortality rate ranges from 8% to 22%. The bleeding pattern of AHA is rather different from that of congenital hemophilia A. Indeed, most patients with FVIII autoantibodies develops hemorrhages into the skin, muscles or soft tissues, and mucous membranes (e.g., epistaxis, gastrointestinal and urologic bleeds, retroperitoneal hematomas, postpartum bleeding), whereas hemarthrosis, a typical feature of congenital factor VIII deficiency, are uncommon [2,3,4,5]. The hemorrhages are often serious or life threatening as the disease may dramatically manifest with excessive bleeding following trauma or surgery or by cerebral hemorrhage. The incidence of AHA increases with age, being a very uncommon condition in children. Indeed, the incidence in children younger than 16 years has been estimated to be approximately 0.045 per million/year compared to 14.7 per million/year in elderlies aged older than 85 years. However, it is also likely that the incidence of this autoimmune disorder is significantly underestimated, especially in elderly patients [6]. In approximately 50% of cases, FVIII autoantibodies occur in patients lacking any relevant concomitant disease, while the remaining cases may be associated with postpartum period, autoimmune diseases, underlying hematologic or solid cancers, infections, or use of medications and dermatologic diseases (pemphigus and psoriasis) [5,7,8]. Approximately 5% of AHA cases occur in relation to pregnancy, usually post-partum [3,5] (Table 1).

**Figure 1:**
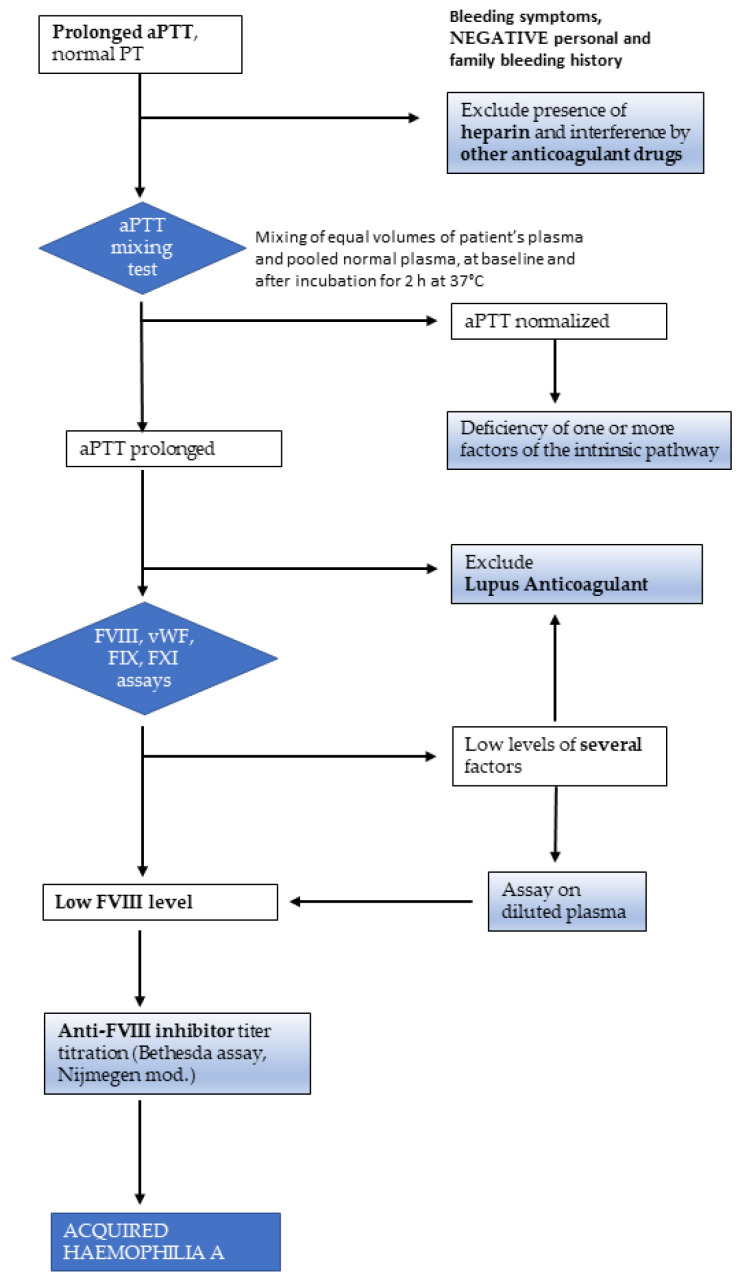
Diagnostic algorithm for acquired haemophilia A. Measurement of FXII level is not indicated in the presence of bleeding symptoms, because even severe deficiencies of this factor, which causes prolongation of the aPTT, are not associated with bleeding tendency. aPTT: activated partial thromboplastin time; PT: prothrombin time; FVIII: factor VIII; FIX: factor IX; FXI: factor XI; vWF: von Willebrand factor.

More recently, the incidence of AHA has risen to 6 inhabitants per million, probably due to higher awareness of the disease and the usage of novel drugs reducing immunotolerance and stimulating autoimmunity against FVIII. Initial testing should include an activated partial thromboplastin time (aPTT) and prothrombin time (PT). AHA patients have an isolated prolonged aPTT, and initial screening with a 1:1 mixing study with normal pooled plasma does not correct the prolonged clotting time (Figure 1). PTT prolongation is quite aspecific and can occur in several conditions, such as in the presence of interfering substances (e.g., heparins), and lupus anticoagulants must be ruled out and FVIII activity assessed. FVIII activity is <1% in approximately 50% of cases and less than 5% in 75% of cases [16]. The dilute Russell viper venom test (DRVVT) can be used for lupus anticoagulant testing and is not typically affected by FVIII inhibitors. Chromogenic FVIII activity assays are not sensitive to lupus anticoagulants [17,18,19]. Bleeding manifestations, PTT prolongation and abnormalities of mixing tests together with low levels of FVIII are highly suggestive for AHA. If FVIII level is low and other tests are negative, quantification of the inhibitor titer via Bethesda assay should then be conducted. It should be noted that patients may have concurrent lupus anticoagulants and FVIII inhibitors. Thus, identification of a lupus anticoagulant in a patient with a newly prolonged aPTT and bleeding manifestations does not entirely rule out the presence of an acquired inhibitor to FVIII [20,21].

The inhibitor titer equals the reciprocal of the plasma dilution that results in 50% inhibition of FVIII in normal plasma after incubation for 2 h at 37 °C [22]. Inhibitor titers are measured in Bethesda units (BU), where 1 BU is equal to the amount of antibody that neutralizes 50% FVIII activity. Although quite accurate for type I inhibitors that display linear kinetics, autoantibodies in AHA can display type II kinetics, which have some residual FVIII activity; thus, the Bethesda assay may underestimate the titer in the presence of type II inhibitors [23,24,25]. Sensitivity and specificity of the Bethesda assay is improved by the Nijmegen modification (buffering the normal plasma) and heat inactivation of the patient’s plasma prior to assessment [26,27]. Enzyme-linked immunosorbent assays (ELISA) can be used to diagnose FVIII antibodies, but these cannot distinguish neutralizing capacity and are seen to some degree in the normal population (see below). Finally, if recombinant porcine FVIII (rpFVIII) is a therapeutic option, then a Bethesda assay specific to rpFVIII should be considered as it may help guide treatment decisions (see hemostatic therapies section) [16]. Due to its rarity, the disorder occurs almost always unexpectedly and is often unrecognized [5].

Immunotherapy is a recent therapeutic option that targets the patient’s self-tolerance against tumor cells [28]. Immune checkpoint inhibitors (ICIs) have changed the treatment and the outcomes of patients with different type of malignancies by activating pathways that regulate the immune response [29].

**Figure 2:**
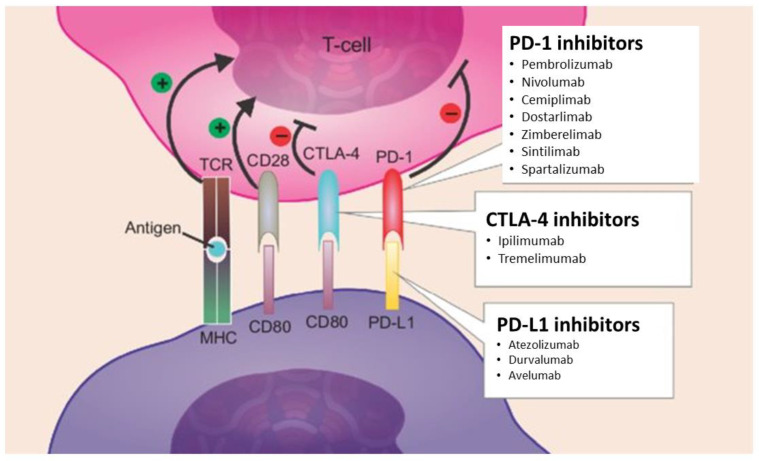
Mechanism of action of immune checkpoint inhibitors. Notes: T regs depend on the activity of CTLA-4, PD-1, and PD-L1 to induce immunosuppression. Ipilimumab and tremelimumab are monoclonal antibodies that inhibit CTLA-4, while pembrolizumab, nivolumab, cemiplimab, dostarlimab, zimberelimab, sintilimab, spartalizumab inhibit PD-1 and atezolizumab, durvalumab, avelumab PD-L1. These drugs act by reducing immuno checkpoint activity on a T reg -rich microenvironment, thus diminishing tumor evasion. Abbreviations: T regs, regulatory T-cells; TCR, T-cell receptor; MHC, major histocompatibility complex.

There are currently six ICIs approved for treatment of malignant melanoma, non–smallcells lung carcinomas, head and neck squamous-cell carcinomas, gastric carcinomas and solid tumors with high microsatellite instability or mismatch-repair protein deficiency [30]. ICIs induce blockade of PD-1 or its ligand, programmed cell death ligand 1 (PD-L1), thus increasing antitumor immunity by blocking intrinsic down regulators of immunity. The immune response is normally regulated by the binding of CD80/CD86 and PD-L1 to their conjugate receptors, cytotoxic T-lymphocyte–associated-4 (CTLA-4) and PD-1 expressed on the surface of cytotoxic T cells. ICIs block these ligand–receptor interactions, releasing an immune response toward the tumor (Figure 2) [31].

**Figure 3:**
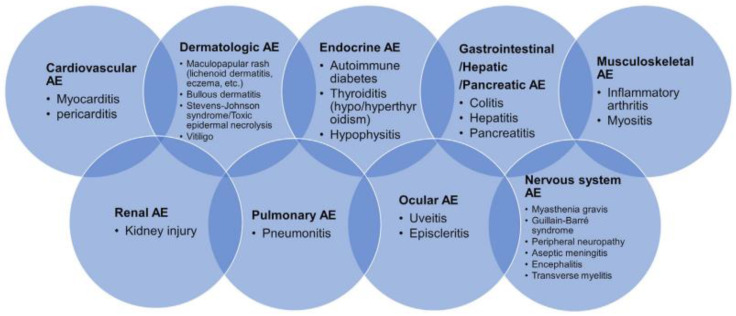
Immune-related adverse events (irAEs) in various organs. National Comprehensive Cancer Network notices irAEs in multiple organs.

Because their therapeutic effects are mediated by enhancing the activity of the immune system, autoimmune-related adverse effects can be seen in up to 80% of patients during and after treatment. Common immune-related adverse events, including dermatological, gastrointestinal, pulmonary and endocrine, are well known (Figure 3).

However, hematologic toxicities have been poorly described, partially because of their uncommon nature but possibly also because of lack of recognition [32]. Most frequent events are represented by aplastic anemia, neutropenia, hemolytic anemia, pure red cell aplasia, autoimmune thrombocytopenia, immune mediated thrombosis. A rare hematologic immune-related adverse event is AHA [33,34,35].

Two PD-1 inhibitors, nivolumab and pembrolizumab, are associated with significant improvement in both progression free survival and overall survival if compared to classical chemotherapy schemes in metastatic melanoma.

Here we report two cases of AHA developed during immunotherapy for advanced melanoma. One was secondary to treatment with nivolumab and one was secondary to pembrolizumab. Both patients were treated with activated FVII (Novoseven^®^, Novo Nordisk, Bagsværd, Denmark) as hemostatic treatment and with rituximab for the eradication of antibodies anti FVIII. In the last few years these drugs have significantly improved the therapeutic armamentarium for the management of AHA. Indeed, while FVIIa has proven to be an effective and safe tool for the treatment of acute bleeding related to FVIII autoantibodies, rituximab is a promising alternative for the autoantibodies’ elimination and the restoration of the normal hemostasis.

## 2. Case 1 Presentation

A 67-years-old man was diagnosed with metastatic melanoma in 2019. After the development of metastasis in bones and lymph nodes, despite the presence of a BRAF-V600E mutation (nucleotide 1799 T > A; codon GTG > GAG) he received four consecutive courses of treatment with chemotherapy (fotemustine and cyclophosphamide) and sorafenib. In 2021 his disease progressed with metastasis in liver, bones, and intra-abdominal lymph nodes. At this time, nivolumab as an intravenous monotherapy infusion of 240 mg every 2 weeks was started. Few days before the fourth injection, the patient claimed macroscopic hematuria. The hemoglobin level was 7.6 g/dL. The laparoscopic resection of bladder nodes showed metastasis. Laboratory investigations revealed a markedly prolonged activated partial thromboplastin time (aPTT, 99 s; ratio 2.7), not corrected with normal plasma (1:1) after a 2-h incubation (prothrombin time (PT) was in normal range). At the same time, heparin contamination, lupus anticoagulants, and other autoimmune diseases were excluded. After investigation, the isolated prolongation of activated partial-thromboplastin time was referred to the presence of a factor VIII inhibitor (factor VIII level <1%; inhibitor titer 26 Bethesda units). Since the patient had reported no personal or family history of hemophilia A (with previous normal aPTT after melanoma diagnosis and before nivolumab treatment) and no history of bleeding during previous surgeries, these findings strongly suggested the diagnosis of AHA, as revealed by hemorrhagic bladder metastasis.

**Figure 4:**
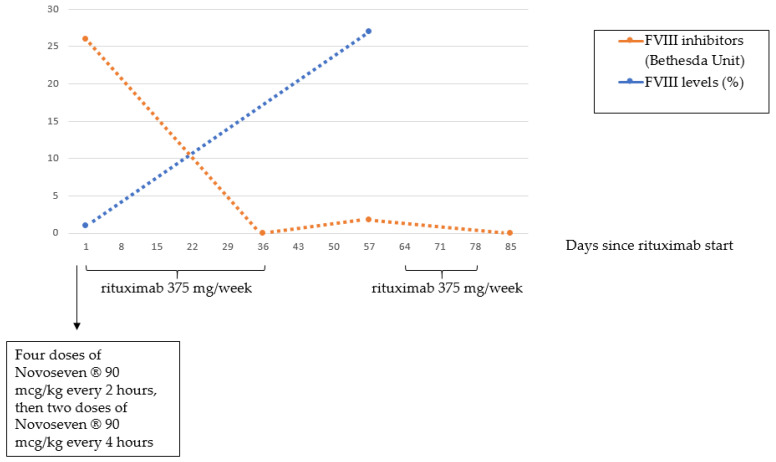
Time-course of FVIII inhibitor titer and FVIII levels in relation with Novoseven^®^ and rituximab administration.

The patient initially received prednisone at a dose of 1 mg/kg body weight orally given and, due to the simultaneously acute bleeding stage, treatment with FVIIa (Novoseven^®^, Novo Nordisk, Bagsværd, Denmark) was also started at a dose of 90 mcg/kg every 2 h (4 doses) and then every 4 h for a total of 6 doses. Despite the low FVIII inhibitor title at the time of the diagnosis, no response to corticosteroids was obtained. Even though the prolonged treatment with FVIIa, the bleeding persisted. The patient was then sent to our Institution, where treatment with rituximab was initiated at a dose of 375 mg/sqm weekly (6 doses, total duration of 6 weeks) in combination with prednisone. After the fifth dose, FVIII inhibitor was undetectable, aPTT normalized and bleeding stopped. For these reasons, prednisone was slowly tapered, but, at +57 days from the start of rituximab, aPTT was found again prolonged (40 s; ratio 1.4) and FVIII levels reduced (27%) with 1.7 BU, without any new hemorrhagic manifestation. Prednisone was then reintroduced at the dose of 1 mg/kg and rituximab was given for 3 additional infusions, obtaining the normalization of aPTT and the disappearance of the inhibitor a week after the eighth rituximab infusion. (Figure 4) The patient stopped prednisone therapy at +150 days from the start of rituximab without clinical signs of bleeding and normal clotting tests. At the last follow-up (+200 days) the patient was still in clinical and laboratory complete remission. Overall, rituximab infusions were well tolerated, without evidence of infusion and/or late reactions. Finally, no infections have been reported so far.

## 3. Case 2 Presentation

**Figure 5:**
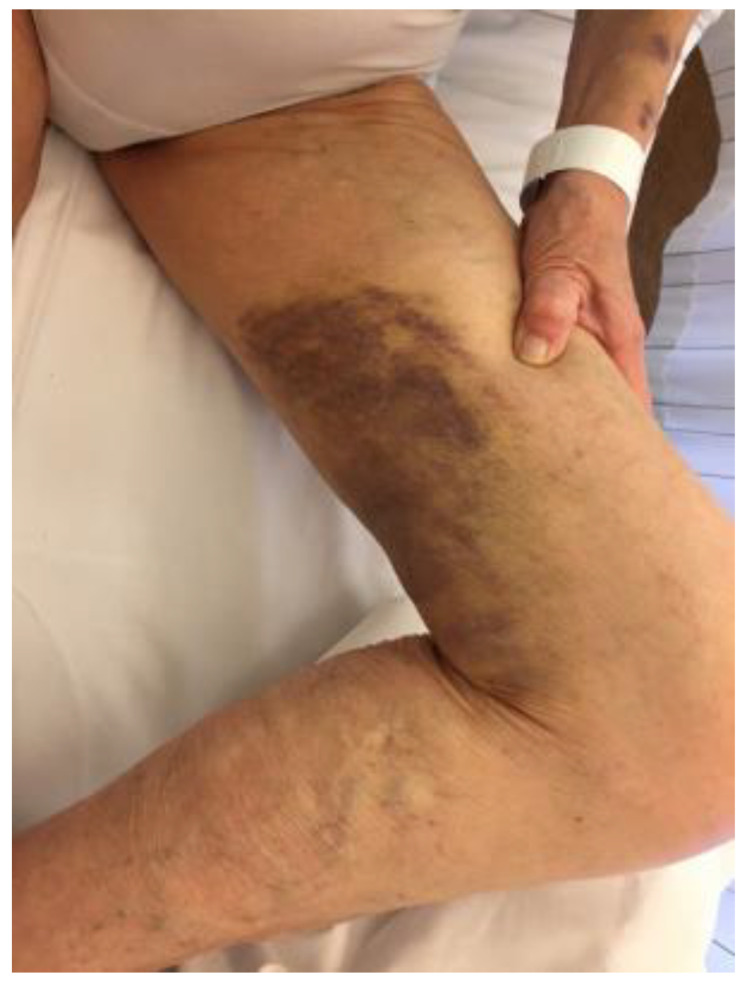
Extensive hematoma on the left upper leg.

A 70-years-old male, with a negative history of prior bleeding diathesis presented with easy bruising and large cutaneous hematomas on both arms, left upper leg (Figure 5).

The patient was followed up in the Dermatology Department for a right ankle BRAF-V600E mutation (nucleotide 1798 G > A; codon GTG > ATG) melanoma and bone metastasis and received first line treatment with pembrolizumab 10 mg/kg every 2 weeks. After 7 injections of the drug the patient complained of easy bruising, calf pain, and progressive asthenia. He was then referred to the hemophilia treatment center for further investigations to detect the cause of bleeding. Previous exams showed normal aPTT after melanoma diagnosis and before pembrolizumab treatment. Physical examination showed multiple large hematomas and a swollen painful left calf muscle, suspected for muscle bleed. In addition, several enlarged inguinal lymph nodes were noted. Laboratory tests showed an aPTT of 70 s, ratio 2.3 and a normal prothrombin time. A mixing experiment with 1:1 patient: normal plasma revealed no normalization of the aPTT (53 s; ratio 1.74). The determination of individual intrinsic coagulation factors showed a FVIII:C activity <0.01 U/mL while vWF antigen and activity were normal. The titer of anti-FVIII antibodies was 71 BU, thus confirming the diagnosis of AHA. The patient was successfully treated with activated FVIIa. Immunosuppressive treatment began with prednisone in a dose of 1 mg/kg and subsequently with rituximab 375 mg/mq weekly (6 doses, total duration of 6 weeks), according to general guidelines due to high titer of antibodies anti FVIII > 20 BU and FVIII < 1 IU/dL. This ended in a gradual decrease of the anti-FVIII titer, the rise of FVIII level and the improvement of the bleeding phenotype. Later the patient was followed-up for 4 years without recurrence of AHA.

## 4. Discussion

AHA is a rare bleeding disorder caused by acquired autoantibodies against FVIII. Soft tissues’ bleeding manifestations are often severe and may occur spontaneously or after minor trauma.

Given the association between cancer and AHA, we cannot rule out that AHA of the above-mentioned patients depend on the associated malignancy. However, the association with melanoma has not been reported [36], and the aPTT exam were normal before beginning of ICIs therapy. Nevertheless, the tight association between the use of PD1/PDL1 inhibitors is strictly connected with the development of autoantibodies anti F VIII. Moreover, genetic investigations have associated polymorphisms in CTLA4, non-hemophilic FVIII gene variants, and human leukocyte antigen (HLA) DRB1*16 and DQB1*0502 with AHA [37,38,39,40].

An unexplained prolongation of the aPTT, not corrected by the in vitro addition of normal plasma (mixing test), is the typical laboratory feature of AHA, and bleeding complications complete the clinical picture of AHA. Again, FVIII level is reduced and the presence of an inhibitor is revealed by Bethesda assay. The fundamental aspects of the therapeutic strategy in patients with AHA are the treatment of acute bleeding episodes and the long-term eradication of the autoantibody [16]. In addition, the treatment of the possible associated disease is fundamental, and, in some cases, it will lead to the disappearance of the inhibitor. Acute bleeding is managed through normalization of factor VIII level. Despite some authors recommend choosing hemostatic treatment, such as recombinant activated FVII (rFVII, Novoseven^®^, Novo Nordisk, Bagsværd, Denmark) and the activated prothrombin complex concentrate (APCC, FEIBA^®^; Baxter Healthcare, Westlake Village, CA, USA), according to the Bethesda assay (<5 BU or >5 BU, respectively), some considerations are needed. As reviewed in detail by Tiede et al. [16], differently from congenital hemophilia, inhibitors in acquired hemophilia do not follow a log-linear dose–response relationship, which is the basis for quantification in Bethesda assay. In addition, an analysis of data from the EACH2 registry demonstrated that bypassing agents are more effective than FVIII infusion [41]. Human FVIII, porcine FVIII, and desmopressin are also used. Immunosuppression is the mainstay to obtain the inhibitor eradication. Steroids alone or combined with cytotoxic agents (i.e., cyclophosphamide, azathioprine, vincristine, or combination therapy), are also frequently used. The combined treatment of steroids with cyclophosphamide is able to eradicate inhibitors in approximately 70% of patients with AHA. The monoclonal antibody rituximab is a chimeric antibody targeting CD20 antigen on B-cell surface [42]. It has been reported to be effective in eradicating the inhibitors in AHA and 172 patients with this condition have been treated so far, including the present cases [43]. Rituximab has been used alone or in combination with other immunosuppressive drugs, such as steroids and cyclophosphamide, as salvage or first line-therapy. Overall, 157 patients (91%) showed a response, with 146 patients (85%) achieving complete response and 20 patients (12%) partial response. Five patients (3%) did not respond to rituximab therapy. The dose of rituximab infused in almost all cases was 375 mg/sqm, as in the lymphoma treatment. However, a lower dose has also been used (100 mg). The number of rituximab infusions was very variable (from a single low-dose to 12 standard doses). In addition, in most cases, 4 standard doses were the applied schedule and the time to response was also heterogeneous (from 1 week to more than one year). Re-treatment with rituximab was generally effective. Overall, the administration of rituximab in AHA was well tolerated with very few infusions side effects, and no infectious complications have been reported so far.

A treatment algorithm proposed by Tiede et al. for first-line therapy for patients with AHA consisted of the association of prednisone (1–2 mg/kg daily) alone or in combination with cyclophosphamide (1–2 mg/kg daily), while rituximab plus corticosteroids therapy should be reserved for second-line treatment or may be indicated as first line in patients with high titer of antibodies anti FVIII > 20 BU and FVIII < 1 IU/dL [16,43].

## 5. Conclusions

Anti-CD20 monoclonal antibody rituximab is increasingly used to treat autoimmune disorders. In the case of AHA, its definitive role in eradicating inhibitors (first or second-line, high or low inhibitor titers, older and/or frail patients for whom corticosteroid and cytotoxic therapy are unsuitable) requires further evaluation. However, randomized-controlled trials are very hard to design because of the rarity of AHA. Furthermore, additional data must be acquired to optimize the use and to compare the efficacy of this drug to other treatments. Current evidence confirms that rituximab should be considered as part of the therapeutic armamentarium of AHA even if secondary to ICIs treatment.

## Figures and Tables

**Table 1:** Demographic and clinical characteristics of patients with acquired hemophilia A in large registry studies. Note: data reported as n (%) or median (range). Abbreviation: NR, not reported.

Study	Delgado [1]	Green [9]	Collins [10]	EACH2 [11]	Tay [12]	Borg [13]	Huang [14]	GTH-AH [15]
Patients (n)	234	215	172	501	25	82	65	102
Age (years)	64 (8–93)		78 (2–98)	74 (62–80)	78 (27–99)	76.7 (25–103)	64 (18–94)	74 (26–97)
Inhibitor (BU/mL)	10 (0.9–32,000)		13 (4–38)	12.8 (4.2–42.5)	11 (1.2–460)	16.1 (1–2800)	19.4 (0.74–2414)	19 (1–1449)
Male (%)	45	53	43	53	48	61	64	58
Underlying disorder								
Idiopathic	135 (57.7)	82 (43.6)	95 (63.3)	260 (51.9)	19 (79)	45 (54.8)	34 (52)	68 (67)
Malignancy	43 (18.4)	12 (6.4)	22 (14.7)	59 (11.8)	1 (4)	16 (19.5)	8 (12)	13 (13)
Autoimmune	22 (9.4)	32 (17.0)	25 (16.7)	67 (13.4)	3 (12)	12 (14.6)	4 (6)	20 (20)
Post-partum	34 (14.5)	13 (7.0)	3 (2.)	42 (8.4)	2 (8)	6 (7.3)	3 (5)	5 (5)
Infection	NR	NR	NR	19 (3.8)	NR	NR	NR	NR
Dermatologic	NR	8 (4.3)	5 (3.3)	7 (1.4)	NR	NR	3 (5)	NR
Drugs	NR	10 (5.3)	NR	17 (3.4)	NR	NR	11 (17)	NR
Other	NR	21 (16.5)	NR	58 (11.6)	NR	NR	2 (3)	NR

## Data Availability

The study data will be made available upon request to the corresponding author.
